# Adolescent boys’ experiences of stress – a focus group study

**DOI:** 10.1186/s40359-024-02076-y

**Published:** 2024-10-19

**Authors:** Manuela Schmidt, Erika Hansson

**Affiliations:** 1 Department of Quality Improvement and Leadership, School of Health and Welfare, Jönköping University, Jönköping, Sweden; 2 Faculty of Education, Kristianstad University, Kristianstad, Sweden

**Keywords:** Adolescence, Mental health, Peers, Qualitative methods, Stress

## Abstract

**Background:**

The number of adolescents reporting that they are stressed has more than doubled among both boys and girls. Most focus is given to girls because they typically not only report higher levels of stress but also feel stressed more frequently than boys do. However, studies have confirmed that boys’ experience of stressors is the same, implying that genders are equally prone to experiencing stress. Although male and female adolescents appear to experience stress in a similar way, how they cope with these stressors might differ. This study focused on boys’ experiences of stress and how they cope with it.

**Method:**

Eight focus groups were conducted with 39 adolescent boys aged 12–19 years enrolled in four schools in southern Sweden. The data was analysed inductively with conventional content analysis.

**Results:**

The analysis resulted in three categories: *Stress perception – time as a key factor*,* Identifying stressors and levels of stress*, and *Silent struggles and distractions*. The boys had experienced considerable stress in their lives, despite their limited understanding of the concept. Their coping skills focused on engaging in sports or distracting themselves from stressors while relying less on social support from peers, school staff, or family.

**Conclusions:**

Adolescent boys might need assistance in identifying stress and clearly voicing their specific concerns. They should also be provided with spaces that are free from stigmatization and judgement. Parents, teachers, and school nurses should be equipped with the appropriate tools and education on how to discuss stress and mental health in general with adolescent boys to prevent possible negative long-term consequences for both their physical and mental health.

## Background

Swedish adolescents are generally more stressed than a decade ago, and the number of adolescents reporting that they are stressed has more than doubled among both boys and girls [1]. Typical signs of stress among adolescents are headaches, stomach aches, sleep issues and/or difficulties concentrating, avoidance of activities, withdrawal from peers, and increased worry [2]. The prevalence of stress and its consequences, including depression [3], has increased in many countries and age groups over the last few years in particular. In the US, for example, the prevalence of depression has increased by 27% among adolescents in recent years [4]. Girls are singled out because they typically not only report higher levels of stress but also feel stressed more frequently than boys do [5, 6]. A review confirmed that girls in general feel more stressed than boys, however their experience of stressors was the same, implying that the genders are equally prone to experiencing stress [7]. Thus, this study focused on boys’ experiences of stress and how they cope with it.

### Adolescent stressors

Adolescents spend much of their time in school, which plays a fundamental role in their development as well as their physical and psychological well-being [2]. Stress related to schoolwork and academic performance has consistently been identified as a major stressor among adolescents across the world, including in the US (reported by 53.5% of surveyed adolescents, followed by weight [34.8%] and relationships with friends [34.3%]), India, Norway (alongside peer pressure and conflicts between school and leisure activities), Ireland and Scotland (along with uncertainties about the future), and Korea (reported by 54.7% of surveyed adolescents, followed by conflicts with parents [15.2%]) [5, 6, 8–10]. In the latter study, stress severity was very high or high (41%) and around one third of the adolescents experienced depressive episodes [10]. In Sweden, adolescents reported a continuous decrease of general school enjoyment and well-being in school in 2021/2022 compared to a decade ago [1]. High well-being in the school environment and school connectedness are related to improved academic achievement, while low well-being and school connectedness are correlated with increased risk behaviour such as drug and alcohol usage as well as lower self-reported health [1, 11, 12].

A recent study by Jakobsson et al. [13] showed that adolescents differentiate between school stress, everyday stress, and not wanting to miss out on life. Apart from schoolwork, adolescents undergo additional diverse challenges in their everyday lives. For example, adolescents have reported experiencing romantic relationships, safety, and autonomy concerns as stressful [8]. Indeed, not only do adolescents have to cope with puberty and physical change, but they also have to manage the social relationship dynamics with their peers and their home life. The socioeconomic status and financial conditions of parents have also been identified as stressors in adolescents’ lives [7]. These everyday stressors can have severe negative impacts on individuals’ health, such as their mental well-being, brain development and academic performance (e.g. [4, 10, 12, 14]). Furthermore, different stressors can have a negative impact on the sleep and emotions of adolescents [13, 15].

### Adolescent coping

Although male and female adolescents appear to experience stress in a similar way [7], how they cope with these stressors might differ. In general, adolescents cope with stressors through internalising behaviours, engaging in leisure activities, and talking to friends [15], as well as through physical activities, religion, and spiritual rites, as shown in a Korean study [16]. However, boys in general cope with stress by distracting themselves, denying their emotions, or forced relaxation. They also engage in approach-oriented and problem-focused coping [5]. Girls, on the other hand, typically cope through avoidance, seeking support, and openly expressing their emotions [5]; they also tend to ruminate [7].

Adolescent coping behaviour may serve as a mediator between stress and health outcomes. Adolescents’ engagement in maladaptive coping can occur over long periods [7]. Since chronic stress can contribute to physical and mental health problems later in adulthood [17, 18], it is crucial to provide adolescents with the appropriate tools and education early on to increase their awareness of stress and its consequences and how to embrace adequate coping skills. Factoring into this capacity building is adolescents’ self-esteem, which plays a central role in stress management and coping abilities. Adolescence is associated with changes in self-esteem, with lower self-esteem potentially triggering stress and maladaptive coping [19]. It is therefore an important factor to consider when developing such tools and educational programmes to increase awareness.

### Theoretical framework

This study utilises the transactional model of stress and coping [20], which conceptualises stress as an exchange between an individual and their environment. According to this model, stress is the result of a perceived imbalance between the demands of a person’s environment and their own available resources to respond to these demands [20, 21]. Specifically, stress is defined as the result of exposure to stimuli appraised as harmful, threatening, or challenging and which sometimes exceeds the individual’s capacity to handle [20, 22]. These stimuli, called *stressors*, may be internal or external and range from common everyday annoyances to life threatening challenges.

*Coping* – “a function of continuous appraisals and reappraisals of the person-environment relationship” – is a response to stressors [23]. Seiffge-Krenke [24] developed Lazarus and Folkman’s model further by adapting it to adolescents. When engaged in coping, adults and adolescents alike are making a cognitive appraisal of a perceived stressor. This appraisal process is impacted by the stressor itself, the person’s internal coping resources and external social resources. In previous studies, stressors that were identified in the adolescents’ everyday lives were mostly external and primarily related to school performance and social relationships. These stressors appear to be relatively universal across the world [25]. According to Seiffge-Krenke’s model [24], adolescents’ relationships with parents, family and peers are considered particularly important external social resources.

At their developmental stage, adolescents may already have adopted certain coping mechanisms that more or less work well for them in the moment. Studies on adolescent coping have revealed that adolescents engage in a variety of coping skills. There are also gender differences in both stress and coping skills. This study focuses on male adolescents because, although they experience similar levels of stress as female adolescents, they tend to perceive their stress as less severe [26]. Thus, the aim of this study was to explore adolescent boys’ experiences of stress and coping. Understanding their experiences is essential for providing appropriate education and promoting early interventions to strengthen coping among adolescents, as well as helping caregivers and the school administration reduce short term stress, thereby mitigating the negative impacts of adolescent stress in adulthood.

## Method

### Participants

Eight schools in southern Sweden were contacted during the spring semester of 2021 to inform the principals of this research project. Of these, four agreed to participate. The schools were both public and private, had varying intake areas and were located in both low- and high-income communities. Certain teachers were chosen as contact persons responsible for distributing the information letters and consent forms to both the students and their caregivers (via the students) in the selected classes. The classes were chosen based on the students’ age (12–19 years). Only male adolescents were included in the study. In total, 39 adolescent boys participated, as shown in Table 1.

**Table 1:** Participants

Focus group	Participants	Year of birth	School
1	1 student	2008	A
2	7 students	2006–2007	A
3	6 students	2005	A
4	3 students	2003–2004	B
5	5 students	2009	C
6	5 students	2009–2010	C
7	5 students	2007–2008	C
8	7 students	2003–2006*	D

*Class with immigrant background

### Data collection

Eight focus groups were conducted between May and November 2022. Each focus group consisted of a varying number of students due to students’ availability. The first focus group was attended by only one student because other presumptive participants had fallen ill. However, we decided to conduct the interview regardless. All focus groups took place at the school premises during lecture time. Since the focus groups were organized by class, the participants in each group knew each other. A semi-structured interview guide was used, ensuring relative consistency in structure and topics while also allowing for flexibility to create a dialogue between participants. The guide consisted of two parts; one part covering topics related to health, food and eating [27]; and the other covering topics related to stress. This study is based on the data collected with the second part of the guide. The questions used were e.g. “What does the word stress mean to you, what is stress?”, “When do you feel stressed?”, “What stresses you?”, or “What do you do to feel less stressed?”. The guide was tested in two pilot focus groups who were not included in the present study; minor adjustments were made to the guide after these focus groups. The focus groups lasted on average 31 min and were transcribed verbatim.

### Analysis

The focus groups were analysed using conventional content analysis [28], which is a suitable method for describing a given phenomenon – in this case, adolescent boys’ stress. The unit of analysis was the focus group transcriptions. Conventional content analysis utilises an inductive approach, deriving categories directly from the data. The analysis process started with the authors immersing themselves in the data by reading and rereading all material to gain an initial understanding of it. Both authors identified and highlighted text that described adolescents’ experiences of stress and its management and compiled a file with these text units. In the next step, the authors condensed the text units by giving each (a) code(s). The codes captured the core of the text unit but remained close to the text. We worked independently in this coding process. Subsequently, the codes were compared and agreed on by both authors. The final codes were abstracted and organised according to similarities and differences into categories. Both authors discussed the categories and agreed on their labelling, description and interpretation. To ensure trustworthiness we used thick, descriptive quotations as representations for our findings and investigator triangulation in the research team [28, 29].

### Ethics

The school principals granted their approval to run the study in their schools prior to data collection. Both caregivers and participating male adolescents were informed about the study through an information letter, which outlined the study’s objectives and procedures and emphasized that participation was voluntary and could be withdrawn at any time without providing a reason. The authors’ contact information was included. Informed consent was obtained from all participants before data collection began. Additionally, informed consent was obtained from caregivers if the adolescents were 16 years old or younger. The study was approved by the Swedish Ethics Review Authority (Dnr 2017994).

## Results

The analysis yielded three categories. Below we describe each category and use rich descriptions combined with quotes from the focus groups (FG1–FG8) to clarify the content and meaning of each category.

### Stress perception – time as a key factor

The first category captures perceptions of the concept of stress as rather limited for the adolescent boys, who mostly discussed the dimension of *time* as a factor in stress, such as time pressure, deadlines, haste due to a lack of time, or not having enough time.

*So*,* it can be… it can be that one maybe has a lot to do. And then it becomes difficult*,* and then you don’t have time*,* and then you can get stressed… One doesn’t have enough time… So*,* stress for me is when I use my time in the wrong way. That I spend my time on things I shouldn’t*,* and then there are more important things for which I then don’t have enough time. (FG1)*

In a few instances, boys perceived stress as a lack of focus or organizational skills. Another perception of stress was when they felt scared about something. However, several boys could not provide a clear definition of the concept and avoided using the word altogether due to its negative connotations.

*So*,* it happens*,* not exactly that you say “Damn*,* now it’s stressful” or something like that*,* like “Oh*,* shit*,* we have this task”*,* so you say things that imply stress*,* even if you don’t use the word “stress”. So*,* using phrases like yeah*,* “We have an assignment due on Friday and it’s Wednesday and none of us have done it*,* ah*,* shit”. And then you think like “Now we kind of have to do it and we have to do it quickly”.* (FG2)

Boys’ limited understanding of stress could be partly explained by the fact that several of them perceived themselves as not having experienced any stressful events. However, the boys in general expressed an understanding of the difference between what they called temporary and *unrealistic* stress – for example when playing videogames – as opposed to more realistic stressful situations.

### Identifying stressors and levels of stress

The second category describes different levels of stress, ranging from experiencing stress infrequently to feeling stressed constantly. One boy said: *“I am very stressed*,* and I feel bad every day”* (FG2). The stressors mentioned by boys were primarily related to school, centring on issues such as incomplete or excessive assignments, homework, upcoming exams, feeling unprepared for tests, falling asleep during tests, tardiness, oversleeping, missing school, giving presentations, struggling to keep up with school subjects, or receiving poor grades.

*1: That was in sixth grade*,* I think*,* when we had just received grades [grades are introduced in the sixth grade in Sweden]. So*,* you sit there thinking*,* “Now we’re going to get grades*,* we’re going to get grades*,* we’re going to get grades.”*

Moderator: *Mmm.*

*1: Thinking …. [very quietly] …. we’re going to get grades.*

*You think about it*,* like*,* on the way to school and on the way home from school. During breaks*,* “We have grades”.*

*2: Yes.*

*3: we have grades! [Laughs]*

*1: I have to do my best in school now.* (FG6)

Boys showed notable self-awareness by recognizing their own role in stressful situations and admitting that they could be due to a lack of prioritizing skills. Some boys forgot topics discussed in class, while others admitted to being lazy and listless and chose only to complete tasks that they found fun. They showed a good understanding of when they were wasting time that would later cause time pressure.

*1: Yes*,* but like M said*,* if you maybe have some kind of assignment to do*,* and you don’t do it*,* you play games or something instead…*.

Moderator: *Yes.*

*2: Waste your time on other things.*

Moderator: *So*,* you’re all aware that you kind of… will become… will be stressed because you spend your time on other things?*

*1: Yes.*

Moderator: *But you still choose to spend your time on those other things?*

*1: Yes.*

*3: Yes.”* (FG2).

Stressful events beyond the school environment were also encountered. Several boys talked about incidents of theft, physical assault, or bullying, all of which were highly stressful situations for them. Additionally, some boys lived in households where severe illness was prevalent or where they had to deal with conflicts among siblings. Some experienced stress due to a fear of crowded places (agoraphobia), while others anticipated a stressful future. A few were also concerned about how they would be perceived by others or not achieving their desired body weight.

*I worry about stress a lot*,* especially about school… managing school*,* having a good job in the future*,* what do I want to do*,* or what… and sometimes I worry about what other people think of me*,* why do they say… what are they saying about me and stuff.* (FG4)

### Silent struggles and distractions

The third category covers boys’ descriptions of how they managed their stress, detailing several different coping strategies. Several boys expressed that they could focus on the task ahead when pressed for time: *“I just kind of sit down and do it”* (FG1). Others turned to sports to alleviate stress, as it helped them shift their focus to other matters. Engaging in sports provided a distraction, allowing them to momentarily forget about their problems and find relief. More unorthodox methods such as cheating also helped some boys to deal with stressful situations.

Many of the boys said that they did not talk about their stressful experiences; it was simply not a topic of discussion. For those engaged in suppressing their thoughts and feelings, it could be due to fear of appearing weak or of being ridiculed by others. Several boys were advised not to cry, especially by their fathers.

Moderator: *Do you talk about stress with each other?*

*1: No.*

*2: No.*

Moderator: *No?*

*2: No.*

Moderator: *Not at all?*

*1: No.*

*3: No.* (FG3)

Several boys stated that they tried to ignore their stress altogether. For others, it had become part of everyday life, and as they got used to it, they expressed that they learned to live with it.

Moderator: *Something you learn to live with?*

*1: Something that’s just there.*

Moderator: *Hm! … It must be tough.*

*1: Like*,* yeah*,* I know the stress is coming and I’m used to it*,* so I can handle it*,* it doesn’t affect how I feel or anything.*

Moderator: *Would you say you’ve learned to handle it?*

*1: Yes. It’s like it doesn’t affect me at all.*

*2: Well*,* that’s probably true*,* what he’s saying*,* that…*.

Moderator: *That you’ve learned…*.

*2: Yes*,* exactly*,* that you’ve learned to cope with it.*

Moderator: *Yes.*

*1: It’s like I just get stressed*,* yeah*,* but it still doesn’t affect your body*,* even though you’re stressed.* (FG2)

The boys held different opinions about talking about their experienced stress. While some stated that talking about it only would make matters worse, others thought that talking was crucial to reach a solution and to feel better. *“If you are stressed and keep it in*,* I actually feel worse because of it. That’s why you need to talk to someone and solve it”* (FG4).

Moderator: *Mmm. Do you wish you could talk more with others?*

*1: No.*

*2: No.*

Moderator: *You don’t feel like that might be a good way to feel better?*

*1: Well*,* if you talk more when you’re feeling bad*,* then you just feel worse.*

Moderator: *Yes.*

*1: I mean*,* if you don’t talk about it*,* you almost forget about it*,* right…* (FG3).

One boy stood out as an exception, as he sought support from a teacher and adopted a more analytical approach to coping with his challenges: “*I often talk to my family*,* my mom*,* and also S*,* my teacher*,* I’ve talked to him several times*” (FG 4). However, in general, the boys’ experiences indicated that the school staff were not a dependable source of support; they had minimal trust in confiding their problems to staff. Those who did choose to talk to someone often relied on a friend or close relative, someone they trusted. Even then, some felt that they were not taken seriously: *“No*,* if I talk to my mom*,* she’ll give me that look ‘Like you know what stress is’ [laughs]… Mom*,* she complains that you don’t know what stress is…”*(FG 3). When comparing themselves to girls, they thought that girls talked with each other about their stress and feelings much more and were more open about it. They also thought that girls experienced more stress than boys.

*1: Even worse.*

Moderator: *Worse?*

*2: That’s the only thing they do*,* sort of.*

Moderator: *Are they stressing or talking about it?*

*1: Both.* (FG9)

*1: They talk too much*,* so that’s why.*

Moderator: *They talk too much*,* so therefore they get stressed?*

*2: I think it’s because they are more open.*

Moderator: *Yes.*

*2: About how they’re doing and such.*

Moderator: *Yes. Do you think it’s positive or negative?*

*2: I think it’s positive.”* (FG8).

Finally, the boys observed that individuals in their immediate environment, such as parents or friends, who were stressed, could inadvertently transfer that stress and its negative consequences to them. Additionally, some boys contemplated the necessity of creating emotional distance from friends who frequently vocalized their stress or problems and engaged in rumination, as they found it detrimental to their own well-being. Instead, the boys favoured a circle of friends who provided support and encouragement, but did not talk about issues with stress. As the discussion deepened, the boys acknowledged that sharing thoughts and feelings with someone, both positive and negative, could have its advantages.

## Discussion

The aim of this study was to explore adolescent boys’ experiences of stress and coping. Overall, the results indicated that boys seem to have a vague understanding of stress and avoid using the term. Acknowledging stress – let alone talking about it – appeared to be seen as a weakness.

### Perceiving stress

Boys’ somewhat vague understanding of stress could partially be explained by the fact that the school curriculum does not cover or discuss stress and related issues. Another avenue worth exploring is that some boys perceived (or at least said they did) not having experienced stress and therefore did not think much about it. However, it is not always that adolescent boys – or people in general – behave exactly as they say. Given adolescents’ comprehension and discussion of explicit stressors, we might conclude that they do experience considerable stress in their lives, despite their basic understanding of the concept. Those findings are well in line with results from other studies (e.g. [7, 10, 15]) and are supported by findings lifting the perspectives of parents, teachers, school nurses and care registers (e.g. [4, 30, 31]).

### Grades at play – decoding school stress

Much of the stress the boys experienced was linked to school performance and grades. This finding aligns with Jakobsson et al. [13], which showed in their study that Swedish adolescents perceive school-related stress differently from other forms of stress. Notably, the role of grading and grades is a significant stressor for the adolescent boys, warranting further scrutiny [32]. This is particularly relevant because the Swedish government is currently considering the possibility of introducing grading at an earlier age, possibly before the current age of 12 years, which was recently lowered from fourteen [33]. However, grades were not the only factor discussed: deadlines and how to get to school in time also gave rise to stress. Academic stress among adolescents could lead to psychiatric problems and decreased quality of life (e.g. [34]). However, the prevalence of academic stress appears to vary depending on the type of education system. In more differentiated and vocationally orientated systems, students are more likely to experience less stress due to e.g. reduced stakes attached to achievements and alleviated competition [35]. Thus, it is important to consider designing education systems that fit the labour market, that trigger more realistic ambitions, that provides students with a manageable workload and fosters positive feelings towards school to alleviate academic stress among adolescents.

### Parents as a source of stress rather than a source of support

Parental stress was also reported as a stressor among the participating boys. These findings are supported by other studies conducted in Sweden and other contexts (e.g. [10, 36]). Not only could parents’ stressful situations spill over to their children, but they could also cause stress for adolescents in other ways. Depending on the school system, parental ambitions, or the family’s financial or socioeconomic situation, parents might exert immense pressure on adolescents to perform well academically.

### Coping at play

The boys presented several ways in which they coped with different stressors. They expressed diverging views on whether it felt better or worse to talk to someone when feeling stressed. Some boys denied feeling stressed altogether and others tried to internalize their emotions, which aligns with earlier research [5, 15]. Still others used distractions, which were identified as a coping strategy in past research [5]. Because repressing emotions does not address the root causes of the stress, it is considered a rather short-sighted form of coping; however, it may sometimes work well, given that adolescence is a particularly turbulent period of life and problems that feel overwhelming one day might not be as overwhelming the next. As McKegney [2] reported, Swedish boys withdrew from peers during stressful situations. While this may be detrimental on the one hand, given that peers can be a great source of support, it may on the other hand be a way to avoid rumination – continually speaking of stress and other negative aspects of life [37]. Several boys stated that they engage in physical exercise to cope with stress. This is in line with earlier research [15, 16] yet opposes the findings of McKegney [2], who reported that adolescents tend to avoid such activities. The boys in this study might have viewed physical activity differently – going to the gym or for a run to “blow off steam” might be a great way of relieving stress, but participating in a soccer team with 20 other boys might not. This approach is supported by Meyer et al. [38] who found that the stress-buffering effect of sports only works in combination with intrinsic motivation. Such intrinsic motivation may be high particularly when choosing activities that make adolescents feel autonomous and self-determined, for example, individual physical activities such as visiting a gym.

Self-esteem, i.e. an individual’s subjective evaluation of one’s worth, and self-efficacy, the ability to consider how effectively one solves different tasks and situations, have shown to be aspects of mental health that contribute to mastery of coping with stressors [39]. Adolescent boys in Sweden have shown to have significantly higher self-esteem than girls (13 years: 43% vs. 74% and 15 years: 44% vs. 68%) and significantly higher self-efficacy as well [13 years: 37% vs. 62% and 15 years: 66% vs. 74%] [1], suggesting that they theoretically have the necessary prerequisites to develop sufficient internal coping resources.

### Who to talk to?

The most accessible sources of support for adolescents experiencing stress in general are parents, teachers, school nurses or school counsellors. However, the boys in this study rarely talked to or confided in such sources. In fact, they outright dismissed talking to a school nurse. The adolescents in our study faced some challenges in trusting school staff and school nurses even though the students were aware of the concept of confidentiality. One explanation might be the large number of students (400–500) that a single school nurse is responsible for in Sweden [40], which might make it more difficult for them to establish a trusting relationship that enables students to be comfortable enough to talk about personal matters, such as their mental health. Further, school counsellors have reported that the relatively ambiguous approach to mental health by schools is an problem in general for them, preventing them from being able to promote students’ mental health and well-being [41].

Although a recent national report revealed that students in Sweden do turn to parents for concerns [1], our study indicates that it is not as natural for boys to do so, as they felt that they were met with judgement and prejudice from their parents (e.g. not being allowed to cry or not being taking seriously), which might increase the risk of discarding this source of support in the long term. Furthermore, parents have overall shown a decrease in coping capacity in regard to the demands of raising children over the last years [4], which could potentially explain why adolescent boys do not find suitable listeners who they can reveal their emotions and thoughts to.

The most obvious source of support for adolescents was peers. However, as our study shows, talking to peers was rarely seen as an option. In general, the boys preferred not to talk about their stress altogether. This could be explained by gender stereotypes about boys not showing weakness, particularly given that mental health problems are still considered a personal weakness across the world ([42], cf. [43]).This also aligns with boys’ perceptions of not wanting to be ridiculed or judged by peers. Paradoxically, the boys wanted to be empowered by peers and would end their relationship with peers who often expressed feeling down, as this could affect their own well-being. Thus, this suggests that neither parents nor peers might represent a good external social resource for coping, as claimed by Seiffge-Krenke’s model of adolescent coping [24]. Instead both parents and peers seem to contribute to the status quo of hegemonic masculinity resulting in boys internalising their emotions and suppressing their desire for compassionate relationships since expressing thoughts, feelings or sharing experiences is seen as sensitive and thus ‘weak’ and not manly [44]. Boys and men remaining on this track have also shown to be less likely to rely on positive coping strategies [44]. Our results however also indicate that, after some discussion, boys can see the benefits of talking to each other about sensitive issues like stress. This suggests they would welcome relationships that validate their authenticity and vulnerability if given the right opportunities to express themselves.

### Consequences

The consequences of experiencing stress and engaging in maladaptive coping can be both short- and long-term for adolescent boys. Certain stressors have shown to impact negatively adolescents’ sleep patterns and emotions [13, 15]. Maladaptive coping might have positive effects in the short-term [45, 46], but over the long-term have negative implications for men’s social and work relationships, psychological well-being, and mental and physical health [17, 18]. If appropriate coping strategies are not learnt in adolescence, it may impact negatively with the individual’s future studies at the university level or particularly demanding events in his work lives, creating stressful situations that one cannot easily cope with or avoid altogether (e.g., not choosing to study at the university level or choosing low demand jobs). A review from 2019 concluded that maladaptive coping increases while adaptive coping decreases as children enter adolescence [47]. The same review also maps the interplay between mal/adaptive coping strategies, developmental changes and learning among children and adolescents. For example, those adolescents capable of utilising several adaptive coping strategies also showed higher levels of positive functioning, self-regulated learning and coping with stress, while adolescents engaging in multiple maladaptive coping strategies tended to use more surface learning and were less effective at dealing with stress. In light of this, our study suggests that boys need to develop adaptive coping skills to handle academic challenges and stress constructively, which will help them reach their educational potential.

### Limitations

This study is not without its limitations. We aimed to be reflexive and sensitive in our data collection process and analysis and showed awareness of the participants’ situations. Yet conducting focus groups with engaging participants is demanding [48], particularly with adolescents. Furthermore, the results indicated a strong focus on stressors found in the school environment. This could be because the data were collected at school or because of the inductive nature of our analysis method, which made us flexible with what experiences that the adolescents wanted to talk about. Thus, this study only focused on stressors experienced in everyday life as described by participants. While we acknowledge the relevance of studies investigating stress and coping in relation to childhood traumas and chronic disease, we felt these sources of stress to be outside of the scope of this study. Finally, we also acknowledge the large impact that age differences and individual characteristics and traits might have on coping skills however our aim did not focus on comparing different age groups and our data was only analysed on group level, which is why those aspects are not covered in this study.

## Conclusions

Adolescent boys appear to have a limited understanding of the concept of stress, but still experienced considerable stress in their lives. The stressors they experienced were primarily concerning school assignments and school life. The boys’ coping skills focused on engaging in sports or distracting themselves from the stressors while social support from peers, school staff, or family was generally discarded. The results suggest that adolescent boys might need assistance in identifying stress and voicing their specific concerns in a clear manner. Such assistance can be provided in any setting, such as in school, during leisure time or at home, with the primary goal being the promotion of boys’ psychological, social, and educational well-being. The boys need to feel that there are spaces for them to express issues with stress, where stigmatization and judgement can be avoided [44]. Further, parents, teachers, and school nurses should be provided with the necessary tools and education on how to discuss stress and mental health in general with adolescent boys to prevent them from suffering from the negative long-term consequences with regards to both physical and mental health. This proposal is strengthened by a recent review on school nurse interventions [49], stressing the need for acute actions for educational and health-promotive purposes that can potentially decease the students’ stress levels and contribute to developing healthy coping strategies.

## Data Availability

The datasets generated and/or analysed during the current study are not publicly available due to the participants of the study not having given written consent for the data to be shared.
